# The Uphill Battle of Performing Education Scholarship: Barriers Educators and Education Researchers Face

**DOI:** 10.5811/westjem.2018.1.36752

**Published:** 2018-03-13

**Authors:** Jaime Jordan, Wendy C. Coates, Samuel Clarke, Daniel Runde, Emilie Fowlkes, Jaqueline Kurth, Lalena Yarris

**Affiliations:** * Harbor-UCLA Medical Center, Department of Emergency Medicine, Torrance, California; † UC Davis Medical Center, Department of Emergency Medicine, Sacramento, California; ‡ University of Iowa, Department of Emergency Medicine, Iowa City, Iowa; § UCLA Ronald Reagan/Olive View, Department of Emergency Medicine, Los Angeles, California; ¶ Oregon Health and Science University, Department of Emergency Medicine, Portland, Oregon

## Abstract

**Introduction:**

Educators and education researchers report that their scholarship is limited by lack of time, funding, mentorship, expertise, and reward. This study aims to evaluate these groups’ perceptions regarding barriers to scholarship and potential strategies for success.

**Methods:**

Core emergency medicine (EM) educators and education researchers completed an online survey consisting of multiple-choice, 10-point Likert scale, and free-response items in 2015. Descriptive statistics were reported. We used qualitative analysis applying a thematic approach to free-response items.

**Results:**

A total of 204 educators and 42 education researchers participated. Education researchers were highly productive: 19/42 reported more than 20 peer-reviewed education scholarship publications on their curricula vitae. In contrast, 68/197 educators reported no education publications within five years. Only a minority, 61/197 had formal research training compared to 25/42 education researchers. Barriers to performing research for both groups were lack of time, competing demands, lack of support, lack of funding, and challenges achieving scientifically rigorous methods and publication. The most common motivators identified were dissemination of knowledge, support of evidence-based practices, and promotion. Respondents advised those who seek greater education research involvement to pursue mentorship, formal research training, collaboration, and rigorous methodological standards.

**Conclusion:**

The most commonly cited barriers were lack of time and competing demands. Stakeholders were motivated by the desire to disseminate knowledge, support evidence-based practices, and achieve promotion. Suggested strategies for success included formal training, mentorship, and collaboration. This information may inform interventions to support educators in their scholarly pursuits and improve the overall quality of education research in EM.

## INTRODUCTION

In recent years, educators have been increasingly challenged to apply evidence-based practice to their teaching. Despite increased production and dissemination of education scholarship, there is still a great need to improve the quality of medical education research and associate educational practices with patient care outcomes.^1^–^9^ Medical educators have reported multiple challenges to their scholarly pursuits, including lack of time, expertise in research methodology, funding, mentorship, collaborators, research support, and reward for their efforts.^10^–^12^ Limited data suggest that lack of time may be the greatest barrier.^11^

A recent workforce study of emergency medicine (EM) educators suggested that while education faculty make up a substantial proportion of a department’s core faculty, departments often lack the full complement of education leadership positions.^13^ Additionally, education faculty must frequently divide their non-clinical time among multiple academic roles.^13^ It was also noted in this study that many departments lack personnel with education research expertise.^13^ Potential interventions have been proposed to address these needs, including building communities of practice to enhance collaboration, increasing opportunities for funding, and devising strategies to gain protected time.^10^ Despite these preliminary studies, how EM educators perceive these barriers and what interventions would be most beneficial in helping to overcome them is still not well understood.

The Council of Emergency Medicine Residency Directors (CORD) Education Scholarship Task Force and CORD Academy for Scholarship in Education in Emergency Medicine recommended that the EM education research community conduct a formal needs assessment to analyze the specific needs of EM educators in order to design and implement interventions to support educators and the field of education research. The objective of this study was to evaluate the perspectives of both core-faculty educators and successful education researchers with regard to the supporting factors and motivators to performing education research, as well as the barriers and their perceived impact, and proposed solutions to assist them in their scholarly endeavors.

## METHODS

### Study Setting and Participants

Core EM education faculty (defined as those individuals whose main academic role is dedicated to the educational mission of the department, including undergraduate medical education, graduate medical education, and faculty development), were identified through email inquiry of individual program leadership (program director and/or program coordinator), program websites, and personal knowledge. We identified successful EM education researchers in one of two ways: (1) by authorship on a manuscript included in *Academic Emergency Medicine*’s “Critical Appraisal of Emergency Medicine Education Research: The Best Publications of [years 2008–2014]”; or (2) designation of “Scholar” from the Association of American Medical Colleges Medical Education Research Certificate at CORD program.^14^–^21^ When an individual belonged to both cohorts, s/he was enrolled in the education researcher arm of the study only. Data collection occurred between October 2015 and December 2015.

This study was deemed exempt by the institutional review board of the Los Angeles Biomedical Research Institute at Harbor-UCLA Medical Center.

**Population Health Research Capsule**

What do we already know about this issue?

Educators face multiple challenges in achieving their education scholarship goals. There is a need to illuminate effective ways to support them and to improve the quality of education research.

What was the research question?

What are educators’ perceptions regarding barriers to performing scholarship and potential strategies for success?

What was the major finding of the study?

Common barriers were lack of time and competing demands. Suggested interventions were training, mentorship, and collaboration.

How does this improve population health?

This information may inform interventions to support educators in their scholarly pursuits and improve the overall quality of education research in emergency medicine.

### Study Design

This was a cross-sectional, mixed-methods needs assessment study, employing a standardized, survey instrument (with validity evidence previously collected) that allowed for free responses suitable for qualitative analysis. Subjects were invited to participate by email and provided with a link to an Internet-based survey, administered through SurveyMonkey®.^22^ Two follow-up email invitations were sent at weekly intervals to non-responders. Informed consent was implied by those participants who chose to click on the survey link. To maximize response rate and include all possible relevant data, completion of all survey questions was not required.

### Instrument Development

The authors developed two surveys, one for each stakeholder group, after literature review and input from members of the CORD Education Scholarship Taskforce to maximize content validity. Instrument development followed established guidelines for survey research.^23^ The surveys consisted of multiple-choice, 10-point Likert scale, and free-response items. To optimize response process validity, items were read aloud among members of the study group and piloted with a small group of reference subjects. Based on results of piloting, we then revised survey items for clarity and brevity. Final versions of the survey instruments are available in Appendix.

### Statistical Analysis

We calculated and reported descriptive statistics for multiple-choice and rating-scale items. Two researchers experienced in qualitative methods, JJ and LY, independently analyzed data from free-response items using a thematic approach. They examined data line by line to identify recurring concepts and then assigned codes, which were further refined into themes using the constant comparative method.^24^ After independent review, the two researchers met to establish a final coding scheme that was applied to all data. Inter-rater agreement was 93.9% and 89.4% for data from core educators and education researchers, respectively. Discrepancies were resolved by in-depth discussion and negotiated consensus.

## RESULTS

### General Results

A convenience sample of 204 core educators and 42 education researchers, from 118/164 (72%) EM training programs in the U.S. and Canada completed the surveys. Of the core educators responding, 159/197 (80.7%) reported performing research, of whom 111 (69.8%) performed research in medical education. Education researchers were highly productive: 19/42 (45.2%) reported more than 20 peer-reviewed education scholarship publications on their curricula vitae. In contrast, 68/197 (34.5%) of core educators had not published any education scholarship in the last five years. Characteristics of participants and scholarly productivity are shown in Table 1.

### Motivators, Rewards, Career Satisfaction

**Figure 1:**
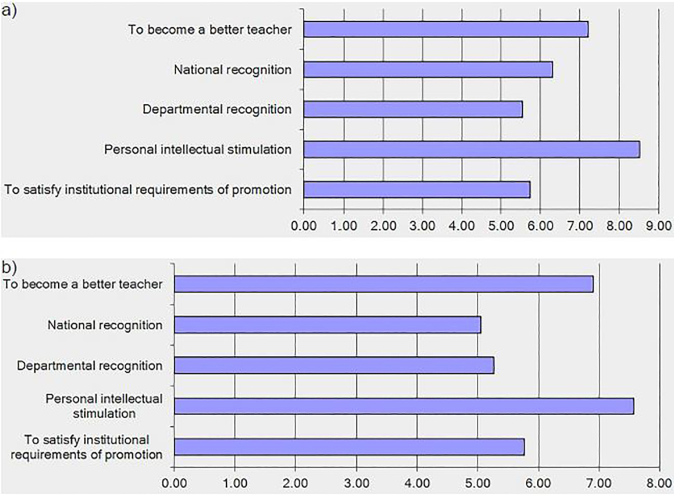
Mean ratings for motivating factors to perform research for a) education researchers and b) core educators (1= Does not motivate me at all; 10= Extremely motivates me).

Our qualitative analysis revealed a number of motivating factors for performing education research in both cohorts. The most prominent of these factors were the desire to disseminate knowledge, support evidence-based practices, meet academic promotion requirements, and personal interest. Results of qualitative analysis for education researchers and core educators are shown in Table 2 and Table 3, respectively. When asked to specifically rate various motivators, education researchers identified personal intellectual stimulation and to become a better teacher as most influential with mean ratings of 8.52 and 7.21 respectively on a 10-point scale (Figure 1). Core educators also rated these factors highest with mean ratings of 7.57 and 6.91 respectively (Figure 1).

**Figure 2:**
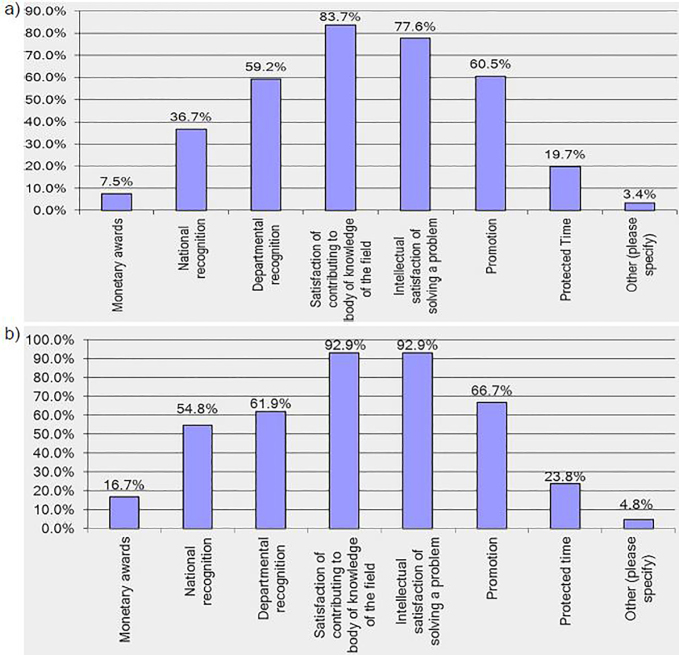
Response rates* for rewards of performing research for a) education researchers and b) core educators. *Participants were instructed to select all options that were applicable, and so results may total more than 100%.

The most common rewards education researchers reported include a sense of accomplishment by contributing to the body of knowledge of the field (39/42; 92.9%) and intellectual satisfaction from solving a problem (39/42; 92.9%) (Figure 2). Core educators also reported rewards of satisfaction of contributing to the body of knowledge of the field (123/147; 83.7%) and intellectual satisfaction of solving a problem (114/147; 77.6%) (Figure 2). Education researchers were satisfied with their achievements in education research and their overall careers, with mean ratings of 7.02 and 8.22 respectively, and felt that performing research contributed positively to their career (mean rating 7.14). Core educators were also satisfied with their careers with a mean rating of 7.62, but less satisfied with their achievements in education research with a mean rating of 4.54. Teaching was the most prominent contributor to career satisfaction for core educators (Table 3).

### Barriers and Challenges

**Figure 3:**
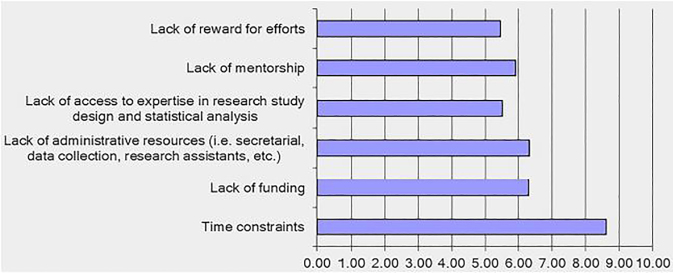
Mean impact ratings of barriers for core educators (1= Does not impact me at all; 10= Greatly impacts me).

Lack of time was the greatest barrier for core educators, with mean rating of 8.61 on a 10-point scale (Figure 3). Core educators reported spending the majority of their time on clinical duties, with mean hours per week of 21.95 ± 10.90, followed by administrative duties 17.53 ± 10.38, teaching 7.58 ± 5.62, research 3.6 ±4.30, and other scholarly work 3.91 ± 3.51. Ideally, core educators would prefer to spend less time on clinical and administrative duties and more time on teaching, research, and other scholarly work. Desired mean hours/week include 18.42 ± 8.45 on clinical duties, 11 ± 7.38 on administrative duties, 9.61 ± 5.91 on teaching, 6.92 ± 5.16 on research, and 4.81 ± 3.41 on other scholarly work. The most prominent challenges for core educators and education researchers were lack of time and competing demands (Tables 2 and 3).

Core educators also cited lack of methodologic expertise as a major barrier. Approximately half of responding core educators (91/183; 49.7%) reported having a mentor. Major themes regarding the positive impact a mentor had on their ability to perform education scholarship for core educators included motivation and training. It should be noted, however, that a contrasting major theme identified was that the core educator’s mentor did not impact this area at all (Table 3). The major theme regarding reasons educators did not have a mentor was lack of an identifiable candidate.

### Strategies for Success

Education researchers and core educators felt that protected time, a collaborative community/research network, and mentorship would help them achieve their research goals (Tables 2 and 3). Core educators indicated they would like to acquire more skills in research study design (112/183; 61.2%), qualitative analysis (88/183; 48.1%), scientific writing (91/183; 49.7%), and quantitative analysis (77/183; (42.1%). The preferred formats for learning skills in medical education research were an online longitudinal course or a longitudinal faculty development course offered at their home institution with 65/181 (35.9%) and 61/181 (33.7%) selecting these options. Less-preferred formats included a daylong session at a professional society national meeting (25/181; 13.8%) or an advanced degree (21/181; 11.6%). Major themes regarding advice from both stakeholder groups to those wishing to become more involved in research included obtaining formal training, finding collaborators, and securing mentorship (Tables 2 and 3).

## DISCUSSION

Critics of medical education research often cite a lack of researcher training and expertise as a key barrier to successful scholarship. While this was identified as a major barrier by our core educator stakeholder group, it is important to note that even formally trained, successful education researchers experienced challenges in this field similar to those who were untrained. In this study, both successful education researchers and core educators identified barriers consistent with prior literature.^10^–^12^ These include barriers that are intrinsic to the researcher such as time constraints and lack of formal research training; extrinsic factors such as lack of funding, lack of research resources, collaborators, mentorship, and leadership support; and barriers inherent to this type of scholarship such as challenges with learners as a study population and perceived futility. The fact that we saw a great deal of overlap between these two stakeholder groups suggests that these barriers may exist regardless of researcher experience or career stage.

The greatest barrier in this study seemed to relate to time constraints. This is not surprising given prior literature emphasizing the complexity and importance of this barrier as well as a prior EM workforce study demonstrating that educators often play multiple critical academic roles and have less time available for scholarly pursuits compared to other job requirements.^11^,^13^ This previously identified mismatch between actual and ideal distribution of workload may not only negatively impact an educator’s ability to perform scholarship but may also adversely affect career satisfaction and burnout.^13^,^25^

Mentorship was also identified as barrier for core educators: less than half of participants in this group reported having a mentor owing to lack of availability. This may indicate that currently few experts are available to meet the needs of education scholars and/or that those with expertise exist outside the field of EM.^13^ Interestingly, successful education researchers were less likely to cite lack of mentorship as a barrier, but did recognize its importance to those looking to pursue education research. This may be because these individuals had available mentorship in their formative years, which may have contributed to their success. Those educators who did have mentorship identified multiple ways their mentorship positively contributed to their scholarly pursuits. It will be important to continue training interested educators in this area to build a cadre of medical education research experts who can meet the training and mentorship needs of future generations and for would-be scholars to look outside of their institution and/or specialty to find this expertise.

We also found a great deal of overlap between motivators to performing education scholarship between core educators and successful education researchers, which may reflect core values such as an emphasis on life-long learning, creation of community of inquiry, desire to achieve success and contribute positively to the field, and to satisfy job requirements and achieve promotion. In this study educators and successful education researchers reported receiving more intrinsic than extrinsic rewards for performing education research. This is in line with prior literature identifying lack of reward as a barrier to performing education scholarship.^10^

Performing research positively contributed to career satisfaction for education researchers, which is not surprising if this is something that they chose to spend their time on despite identifying multiple barriers. Interestingly, despite being satisfied overall with their careers, core educators were less satisfied with their achievements in research. Since research may impact other factors that educators have identified as positively contributing to their satisfaction in this study, such as variety, sense of accomplishment and the potential to enhance teaching, it would be interesting to explore whether educator career satisfaction could be further enhanced by improving their satisfaction with their research achievements.

Advice from those with experience and success in the field is well aligned with the needs identified by participants in both stakeholder groups, further supporting that these are the areas where resources and support should be targeted. Suggested strategies span multiple levels including addressing needs both intrinsic and extrinsic to the would-be scholar, and barriers specific to the field of education research. The aspect of training and acquiring expertise is an expressed need and also recommended advice from those who have been successful. Core educators specifically seek more methodologic training and would prefer a longitudinal online course or one locally available at their institution. These formats are likely preferred because of accessibility since it has already been demonstrated in this study and others that time is an important issue and workload demands for educators are high.^10^–^11^,^13^ To meet the expressed needs identified in this study and follow advice of those with experience and success in the field, future interventions should target an increase in training opportunities, access to expertise, creation of a cadre of trained medical education research experts to serve as mentors, increased funding opportunities and better research infrastructure, and emphasis on the value of this work to garner leadership support and assist in the development of mechanisms to ensure adequate protected time for educators to be successful in their scholarly endeavors.

## LIMITATIONS

This was a convenience sample and completion of all items on the survey was not required as we desired to include all relevant data. It is possible that we may have failed to capture important information. However, this is a fairly large study and given the broad distribution of programs represented, we expect the perspectives expressed by participants to be representative of the group as a whole. Additionally, as this was a survey study, the results must be considered within the context of limitations inherent to this type of design. Despite these limitations, we still believe this study sheds further light on the barriers educators face in performing education research and illuminates motivators and potential strategies for improvement.

## CONCLUSION

Our study identified multiple barriers, motivators/discouragers, as well as strategies for success in performing education scholarship, which were common to both core educators and successful education researchers. The most commonly cited barriers were lack of time and competing demands. Core educators were interested in attaining new skills in education research through faculty development. Key motivators to perform education research for both education researchers and core educators were the desire to disseminate knowledge, support evidence-based practices, and achieve promotion. Suggested strategies for success included formal training, mentorship, and collaboration. This information may inform interventions to support educators in their scholarly pursuits and improve the overall quality of education research in EM.

## Supplementary Information



## Figures and Tables

**Table 1:** Characteristics of EM core educators and education researchers surveyed with regard to barriers and motivations to conduct research.

	Core educators	Education researchers
Gender		
Male	131/204 (64.2%)	ed27/42 (64.3%)
Female	73/204 (35.8%)	15/42 (35.7%)
Age		
<35 years old	26/204 (12.7%)	3/42 (7.1%)
35–50 years old	143/204 (70.1%)	26/42 (61.9%)
51–60 years old	35/204 (17.2%)	12/42 (28.6%)
>65 years old	0/204 (0%)	1/42 (2.4%)
Academic rank		
Instructor	8/204 (3.9%)	0/42 (0%)
Assistant professor	104/204 (51.0%)	15/42 (35.7%)
Associate professor	62/204 (30.4%)	13/42 (31.0%)
Professor	27/204 (13.2%)	11/42 (26.2%)
Other	3/204 (0.01%)	3/42 (7.1%)
Degrees held*		
MD	187/204 (91.7%)	39/42 (92.9%)
DO	16/204 (7.8%)	0/42 (0%)
MPH	12/204 (5.9%)	1/42 (2.4%)
EdD	2/204 (1.0%)	1/42 (2.4%)
PhD	4/204 (2.0%)	4/42 (9.5%)
Other Master’s degree	28/204 (13.7%)	15/42 (35.7%)
Other	5/204 (2.5%)	3/42 (7.1%)
Current position(s)*		
Chair	3/200 (1.5%)	N/A
Vice chair for education	13/200 (6.5%)	N/A
Director of medical education	11/200 (5.5%)	N/A
Education fellowship director	7/200 (3.5%)	N/A
Program director	55/200 (27.5%)	N/A
Assist./associate program director	72/200 (36.0%)	N/A
Clerkship director	30/200 (15.0%)	N/A
Assistant clerkship director	4/200 (2.0%)	N/A
Simulation fellowship director	4/200 (2.0%)	N/A
Simulation director	16/200 (8.0%)	N/A
Other	36/200 (18.0%)	N/A
Fellowship training		
Yes	56/204 (27.5%)	14/41 (34.1%)
No	148/204 (72.5%)	27/41 (65.9%)
Types of peer reviewed medical education scholarship published*		
Research manuscript	91/197 (46.2%)	39/42 (92.9%)
Non research manuscript	49/197 (24.9%)	24/42 (57.1%)
Online curriculum	37/197 (18.8%)	14/42 (33.3%)
Online lecture/instructional video	25/197 (12.7%)	4/42 (9.5%)
None	68/197 (34.5%)	1/42 (2.4%)
Other	12/197 (6.1%)	5/42 (11.9%)
Number of peer-reviewed education scholarship publications listed on curriculum vitae		
0–5	162/196 (82.7%)	9/42 (21.4%)
6–10	21/196 (10.7%)	6/42 (14.3%)
11–15	5/196 (2.6%)	5/42 (11.9%)
16–20	5/196 (2.6%)	3/42 (7.1%)
>20	3/196 (1.5%)	19/42 (45.2%)
Formal training in research methodology		
Yes	61/197 (31.0%)	25/42 (59.5%)
No	136/197 (69.0%)	17/42 (40.5%)

* Participants were instructed to select all options that were applicable, and so results may total more than 100%.

**Table 2:** Results of qualitative analysis for education researchers.

Question	Major themes	Number of comments	Examples
What factors motivate you to perform research?	Dissemination of knowledge	21	“intellectual stimulation, promotion, contribution to the knowledge of the field” “1) I’m interested in advancing the field; 2) I like to share my knowledge; 3) I want to change the way we are doing stuff to be more evidence/theory based.”
	Support evidence-based practice	20	
	Personal interest	14	
	Intellectual stimulation	11	
	Promotion	10	
What factors discourage you from spending time working on your research projects?	Administrative/education demands	22	“other admin/teaching responsibilities; less of a focus for promotion; less hope that grants will buy-down time” “other competing interest; not enough local research infrastructure; little institutional support” “1) Finding blocks of time to design research projects and collaborate with other faculty; 2) Finding reliable, valid tools for assessing the impact of education interventions”
	Clinical demands	17	
	Lack of time	16	
	Personal/family demands	7	
	Perceived futility	5	
	Lack of research support	5	
What challenges have you encountered in performing education research?	Lack of time	31	“1. Funding opportunities in med educ research often modest limiting ambition of research undertaken. 2. Med ed research tends to focus on problem description and diagnosis and less on development and robust evaluation of potential solutions to improve med education. 3. Linked to this there are often epistemiological battles and silos that hinder the development of interdisciplinary impactful research.” “Significant time burden with residency administration, lack of formal training or great senior role models in education research, lack of departmental infrastructure to help execute nonclinical research” “Lack of departmental/institutional support, lack of monetary support, lack of recognition locally that education is important”
	Lack of funding	18	
	Work not valued/lack of leadership support	14	
	Lack of methodologic expertise	13	
	Lack of access to collaborators	11	
Overall, what do you feel would help you achieve your research goals?	Time	17	“Local recognition of its value, financial support, a community locally that supports this interest” “Easier access to biostatisticians and study design experts” “Funding. Allowing for better interdisciplinary engagement between Med Ed research and other relevant disciplines/fields.” “National guidelines for reasonable clinical duties and protected time for education leadership roles”
	Collaborative community/research network	11	
	Access to expertise	9	
	Funding	9	
	Mentorship	6	
	Research support	6	
What advice would you give an EM educator who wants to become more involved in education research?	Obtain formal training	17	“Cultivate mentors, gain a more formal education in education scholarship and don’t go there unless you love it” “find and cultivate relationships with collaborators outside of your department” “1) Identify an area of interest; 2) Take the time to read the literature of what has been done in that area; 3) Seek a mentor in your department or school to provide constructive feedback during the design phase of your research”
	Find collaborators	14	
	Secure mentorship	14	
	Practice patience and persistence	6	
	Inform yourself of current practices/literature	6	

**Table 3:** Results of qualitative analysis of core educators.

Question	Major themes	Number of comments	Examples
What are the major contributors to your career satisfaction?	Teaching	86	“When I witness my residents become a better clinician, educator, or researcher than I am.” “the people I work with, recognizing I am doing something that matters and is very important to the future of medicine” “Sense of accomplishment, benefit of seeing students/residents develop, excellent group that supports education” “autonomy for creation/innovation; multiple types of activities to do”
	Mentorship	37	
	Professional relationships	35	
	Clinical work	35	
	Sense of accomplishment	21	
	Variety	17	
What challenges have you encountered in performing education research?	Lack of time	47	“Mentorship, methodology, time, lack of people interest in the same things…” “lack of respect from chair and others as to importance or rigor of the research” “mentorship in simulation education research with rigorous methods; interdepartmental and across college collaboration; lack of resources in the institution” “difficult to assess outcomes. IRB hurdles. Lack of funding. Inadequate expert support.”
	Lack of methodologic expertise	41	
	Challenges with learners as study population	24	
	Work not valued/lack of leadership support	22	
	Lack of funding	18	
	Lack of mentorship	15	
	Lack of research resources	13	
What advice would you give to an EM educator who wants to become more involved in education research?	Obtain formal training	35	“Seek good mentorship. Consult someone with methodological expertise and someone with statistical expertise while your study is in the design phase.” “Do a fellowship that emphasizes research methodology and an advanced degree.” “find a department that supports and rewards education research” “partner with nationally active peers”
	Secure mentorship	30	
	Find collaborators	20	
	Access expertise	11	
	Secure protected time	11	
	Gather leadership support	10	
What factors motivate you to perform research?	Dissemination of knowledge	47	“demonstrate best practices and disseminate knowledge to help others” “allows one to make evidence-based decisions regarding education.” “scientific knowledge advancement, improved patient care” “need to “publish or perish”” “Job satisfaction; Required for RRC”
	Promotion	46	
	Personal interest	41	
	Intellectual stimulation	31	
	Job requirements	28	
	Support evidence based practice	23	
	Sense of accomplishment	11	
	Contribution to improvement of healthcare	11	
What factors discourage you from spending time working on your research?	Lack of time	74	“wanting to spend time with kids & friends which have greater value to me, desire to create new educational programs” “stretched too thin, lack of mentorship/help with statistics, research support (personnel)” “Almost anything else I do in my job is easier or more fun. Feeling like I’m pushing a big rock uphill trying to get a research process approved or paper published.” “Publication rejections of projects I have spent countless hours on completing. Competing administrative and clinical duties that take time.”
	Administrative/education demands	55	
	Clinical demands	36	
	Lack of research support	34	
	Perceived futility	16	
	Personal/family demands	13	
Overall, what do you feel would help you achieve your research goals?	Time	61	“Protected time for research. Evidence about successful infrastructures. A method of subdividing education research might enhance collaboration. “ “greater mentorship, networking, accountability, funding (funding would at least tie me to a grant with deadlines, reports, deliverables, etc.)” “more formal education in medical education research beyond the few classes in my masters, having a statistician that I trust, having collaborators / mentors”
	Mentorship	28	
	Expertise	27	
	Research support	23	
	Funding	16	
	Collaborative community	15	
	Leadership support	13	
How has your mentor impacted your ability to perform education scholarship?	Positive impact	18	“Has helped me traverse some of the barriers, questioned my proposals in a thoughtful way and suggested strategies for improvement” “I have one mentor specifically trained as a social scientist in qualitative methodology who is actively impacting my ability to perform research by teaching me various skills (e.g. coding, study design, etc.); I have another mentor with EdD background that assisted me with faculty development and educational research/scholarship, and I have two clinical mentors that connect me to large national networks and communities of practice” “My mentor has been instrumental in all aspects, by teaching me the necessary skills, providing me with opportunities, and continually reviewing my work and giving additional suggestions for improvement” “guidance, accountability, offering ideas I had not considered, motivating me to do the work”
	No impact	18	
	Motivation	19	
	Training	14	
	Resources	14	
	Ideas/innovation	10	
How did you find your mentor?	In department	33	“Through a research conference, an education conference, one from my medical school long ago and one from my current department (my chair)”
	During training	24	
	Collaborative work	10	
Why don’t you have a mentor?	Lack of identifiable candidate	46	“no one locally interested in what I am interested in with expertise more than I have” “Proximity, faculty interested in education at home institution early in careers needing mentorship themselves and more senior faculty have other research interests. Education research feels new, although it has been around for quite a while. Perhaps finally getting credit it deserves as a discipline for advancement in Medicine and Medical Schools?”

## References

[1] Sullivan GM, Simpson D, Cook DA (2014). Redefining quality in medical education research: a consumer’s view. J Grad Med Educ.

[2] McGaghie WC, Issenberg SB, Petrusa ER (2010). A critical review of simulation-based medical education research: 2003–2009. Med Educ.

[3] Cook DA, Beckman TJ, Bordage G (2007). Quality of reporting of experimental studies in medical education: a systematic review. Med Educ.

[4] Cook DA, Levinson AJ, Garside S (2011). Method and reporting quality in health professions education research: a systematic review. Med Educ.

[5] Reed DA, Cook DA, Beckman TJ (2007). Association between funding and quality of published medical education research. JAMA.

[6] Chen FM, Bauchner H, Burstin H (2004). A call for outcomes research in medical education. Acad Med.

[7] Lurie SJ (2003). Raising the passing grade for studies of medical education. JAMA.

[8] Reed DA, Beckman TJ, Wright SM (2009). An assessment of the methodologic quality of medical education research studies published in The American Journal of Surgery. Am J Surg.

[9] Cook DA, Bowen JL, Gerrity MS (2008). Proposed standards for medical education submissions to the Journal of General Internal Medicine. J Gen Intern Med.

[10] Yarris LM, Juve AM, Artino AR (2014). Expertise, Time, Money, Mentoring, and Reward: Systematic Barriers that Limit Education Researcher Productivity – Proceedings from the AAMC GEA Workshop. J Grad Med Educ.

[11] Zibrowski EM, Weston WW, Goldszmidt MA (2008). ‘I don’t have time’: issues of fragmentation, prioritisation and motivation for education scholarship among medical faculty. Med Educ.

[12] Goldszmidt MA, Zibrowski EM, Weston WW (2008). Education scholarship: it’s not just a question of ‘degree.’. Med Teach.

[13] Jordan J, Coates WC, Clarke S (2017). Exploring scholarship and the emergency medicine educator: a workforce study. West J Emerg Med.

[14] Farrell SE, Coates WC, Kuhn GJ (2009). Highlights in Emergency Medicine Medical Education Research: 2008. Acad Emerg Med.

[15] Kuhn GJ, Shayne P, Coates WC (2010). Critical appraisal of emergency medicine educational research: the best publications of 2009. Acad Emerg Med.

[16] Shayne P, Coates WC, Farrell SE (2011). Critical appraisal of emergency medicine educational research: the best publications of 2010. Acad Emerg Med.

[17] Fisher J, Lin M, Coates WC (2013). Critical appraisal of emergency medicine educational research: the best publications of 2011. Acad Emerg Med.

[18] Lin M, Fisher J, Coates WC (2014). Critical appraisal of emergency medicine education research: the best publications of 2012. Acad Emerg Med.

[19] Farrell SE, Kuhn GJ, Coates WC (2014). Critical appraisal of emergency medicine education research: the best publications of 2013. Acad Emerg Med.

[20] Yarris LM, Juve AM, Coates WC (2015). Critical appraisal of emergency medicine education research: the best publications of 2014. Acad Emerg Med.

[21] MERC Program Information https://www.cordem.org/i4a/pages/index.cfm?pageid=3344.

[22] SurveyMonkey www.surveymonkey.com.

[23] Rickards G, Magee C, Artino AR (2012). You can’t fix by analysis what you’ve spoiled by design: developing survey instruments and collecting validity evidence. J Grad Med Educ.

[24] Bradley EH, Curry LA, Devers KJ (2007). Qualitative data analysis for health services research: developing taxonomy, themes, and theory. Health Serv Res.

[25] Rothenberger DA (2017). Physician burnout and well-being: a systematic review and framework for action. Dis Colon Rectum.

