# Supplementary material for: The Uphill Battle of Performing Education Scholarship: Barriers Educators and Education Researchers Face
Source: West J Emerg Med. 2018 Mar 13;19(3):619–29. doi: 10.5811/westjem.2018.1.36752 (PMC5942034; doi:10.5811/westjem.2018.1.36752)
Supplement: Supplementary file 1 [file wjem-19-619-s001.docx]

Appendix. Survey Instruments

Survey for Core Educators

Demographic data:

1. Gender: ­­­­­______Male _______Female ________Other/Prefer not to state
2. Age: _____<35 ______35-50 ______51-65 _____>65
3. Please select the academic rank that best describes your current appointment.

_____ Instructor

_____ Assistant Professor

_____ Associate Professor

_____ Professor

_____ Other: ________________________

1. What degrees do you hold? (Please select all that apply)

­­­ ____ MD

____ DO

____ MPH

____ EdD

____ PhD

____ Other Masters degree

____ Other: ___________________________

1. Do you have fellowship training? Yes/No. If so in what? ___________________________
2. Have you published any of the following types of medical education scholarship in the past 5 years? (Check all that apply)

____ Peer reviewed research manuscript

____ Peer reviewed full-length non research manuscript (excludes letters to the editor, etc.)

____ Peer reviewed curriculum published online (such as MedEdPortal)

____ Peer-reviewed On-line lecture or instructional video (such as AEM PERLs, or other peer-reviewed online educational module)

____ Other: ___________________________

____ None

1. Overall, how many peer-reviewed education scholarship publications do you have listed on your CV?

_____ 0-5

_____ 6-10

_____ 11-15

_____ 15-20

_____ >20

1. Have you completed formal training in research methodology? Please describe the type of training you have completed:______________________
2. What is your **current** position? (If you presently hold more than one position, please check all that apply)

­­_____Chair

_____ Vice Chair for Education

_____ Director of Medical Education

_____Medical Education Fellowship Director

_____Residency Program Director

_____Residency Assistant/Associate Program Director

_____Clerkship Director

_____Assistant Clerkship Director

­­­­_____Simulation Fellowship Director

_____Simulation Director

_____Other: ________________________

1. Please rate the significance of the following barriers to your scholarly pursuits: (1= does not impact me at all, 10= greatly impacts me)

Time constraints

1 2 3 4 5 6 7 8 9 10

Lack of funding

1 2 3 4 5 6 7 8 9 10

Lack of administrative resources (i.e. secretarial, data collection, research assistants, etc)

1 2 3 4 5 6 7 8 9 10

Lack of access to expertise in research study design and statistical analysis

1 2 3 4 5 6 7 8 9 10

Lack of mentorship

1 2 3 4 5 6 7 8 9 10

Lack of reward for efforts

1 2 3 4 5 6 7 8 9 10

Please list any other barriers to your scholarly pursuits: _______________________________

1. How many hours do you spend/week on the following:

_____ clinical duties

_____administrative duties

_____teaching

_____research

_____other scholarly work

1. IDEALLY, How many hours would you like to spend/week on the following:

_____ clinical duties

_____administrative duties

_____teaching

_____research

_____other scholarly work

11. Please rate your overall satisfaction with your career: (1= not at all satisfied, 10 = extremely satisfied)

1 2 3 4 5 6 7 8 9 10

12. What are the major contributors to your career satisfaction? (free text box)

13. Do you perform research? (skip logic if yes go to #14, if no go to #23)

14. Do you perform research specifically in the area of medical education? (skip logic if yes go to 15, if no go to# 17)

15. What challenges have you encountered in performing education research ? Please list at least three. (free text box)

16. What advice would you give to an EM educator who wants to become more involved in education research? [free text box]

17. What factors motivate you to perform research? Please list at least three. (Free text box)

18. Please rate the following factors according to how much they motivate you to perform research? (1= does not motivate me at all, 10 = Extremely motivates me)

To satisfy institutional requirements of promotion

1 2 3 4 5 6 7 8 9 10

Personal intellectual stimulation

1 2 3 4 5 6 7 8 9 10

Departmental recognition

1 2 3 4 5 6 7 8 9 10

National recognition

1 2 3 4 5 6 7 8 9 10

To become a better teacher

1 2 3 4 5 6 7 8 9 10

Other:_________________________________

1 2 3 4 5 6 7 8 9 10

19. What rewards do you experience from performing research? Please check all that apply:

_____Monetary awards

_____National recognition

_____Departmental recognition

_____Satisfaction of contributing to body of knowledge of the field

_____Intellectual satisfaction of solving a problem

_____Promotion

_____Protected Time

_____Other:________________________________

20. What factors discourage you from spending time working on your research projects? Please list at least three. (free text box)

21. Please rate your overall satisfaction with your achievements in education research: (1= not at all satisfied, 10 = extremely satisfied)

1 2 3 4 5 6 7 8 9 10

22. Overall, what do you feel would help you achieve your research goals? Free text

23. Which of the following skills would you like to acquire to assist in your career advancement?

_____Research study design

_____Qualitative Analysis

_____Quantitative Analysis

_____Scientific writing

_____None

_____Other:_____________________________________

23. How much time would you reasonably be able to spend participating in faculty development in medical education research? (free text box)

24. What would be your preferred format for learning skills in medical education research?

_____Day long session at a professional society national meeting

_____Advanced Degree

_____Longitudinal faculty development course offered at your home institution

_____Online longitudinal course

_____Other: _________________________________________

24. Do you have a mentor? Yes/No

25. If Yes, how has your mentor impacted your ability to perform education scholarship? How did you find your mentor? (free text box)

If No, why not? (free text box)

Survey for Education Researchers

1. Gender: ­­­­­______Male _______Female _______Other/Prefer not to state
2. Age: _____<35 ______35-50 ______51-65 _____>65
3. Please select the academic rank that best describes your current appointment.

_____ Instructor

_____ Assistant Professor

_____ Associate Professor

_____ Professor

_____ Other: ________________________

1. What degrees do you hold? (Please select all that apply)

­­­ ____ MD

____ DO

____ MPH

____ EdD

____ PhD

____ Other Masters degree

____ Other: ___________________________

1. Do you have fellowship training? YES/NO. If so in what? ___________________________
2. Have you published any of the following types of medical education scholarship in the past 5 years? (Check all that apply)

____ Peer reviewed research manuscript

____ Peer reviewed full-length non research manuscript (excludes letters to the editor, etc.)

____ Peer reviewed curriculum published online (such as MedEdPortal)

____ Peer-reviewed On-line lecture or instructional video (such as AEM PERLs, or other peer-reviewed online educational module)

____ Other: ___________________________

____ None

1. Overall, how many peer-reviewed education scholarship publications do you have listed on your CV?

_____ 0-5

_____ 6-10

_____ 11-15

_____ 15-20

_____ >20

1. Have you completed formal training in research methodology? Please describe the type of training you have completed:______________________
2. What is your **current** position (If you presently hold more than one position, please check all that apply)?

_____ Chair

_____ Vice Chair for Education

_____ Director of Medical Education

_____Medical Education Fellowship Director

_____Residency Program Director

_____Residency Assistant/Associate Program Director

_____Clerkship Director

_____Assistant Clerkship Director

­­­­_____Simulation Fellowship Director

_____Simulation Director

_____Research Director

_____Other: ________________________

1. What challenges have you encountered in performing education research? Please list at least three. (free text box)
2. What advice would you give an EM educator who wants to become more involved in education research? (free text box)
3. What factors motivate you to perform research in medical education? Please list at least three. (free text box)
4. Please rate the following factors according to how much they motivate you to perform research? (1= does not motivate me at all, 10 = Extremely motivates me)

To satisfy institutional requirements of promotion

1 2 3 4 5 6 7 8 9 10

Personal intellectual stimulation

1 2 3 4 5 6 7 8 9 10

Departmental recognition

1 2 3 4 5 6 7 8 9 10

National recognition

1 2 3 4 5 6 7 8 9 10

To become a better teacher

1 2 3 4 5 6 7 8 9 10

Other:_________________________________

1 2 3 4 5 6 7 8 9 10

1. What rewards do you experience from performing research in education? Please check all that apply:

_____Monetary awards

_____National recognition

_____Departmental recognition

_____Satisfaction of contributing to body of knowledge of the field

_____Intellectual satisfaction of solving a problem

_____Promotion

_____Protected Time

_____Other:________________________________

1. What factors discourage you from spending time working on your research projects? Please list at least three. (free text box)

16. Please rate your overall satisfaction with your career: (1= not at all satisfied, 10 = extremely satisfied)

1 2 3 4 5 6 7 8 9 10

17. Please rate your overall satisfaction with your achievements in education research: (1= not at all satisfied, 10 = extremely satisfied)

1 2 3 4 5 6 7 8 9 10

18. Please rate the degree to which performing research contributes to your career satisfaction: (1 = almost none, 10 = Very much)

1 2 3 4 5 6 7 8 9 10

19. Overall, what do you feel would help you achieve your research goals? (Free text)
